# Pharmacogenomics and Big Data in medical oncology: developments and challenges

**DOI:** 10.1177/17588359241287658

**Published:** 2024-10-18

**Authors:** Loredana G. Marcu, David C. Marcu

**Affiliations:** UniSA Allied Health and Human Performance, University of South Australia, Adelaide, SA 5001, Australia; Faculty of Informatics and Science, University of Oradea, Oradea 410087, Romania; Faculty of Electrical Engineering and Information Technology, University of Oradea, Oradea, Romania

**Keywords:** big data, chemotherapy, drug–gene interaction, normal tissue effects, personalized therapy

## Abstract

Medical oncology, through conventional chemotherapy as well as targeted drugs, remains an important component of cancer patient management, particularly for systemic disease. Despite advances in all areas of medical oncology, certain challenges persist in the form of drug resistance and severe normal tissue toxicity. These unwanted effects can be counteracted through a patient-tailored treatment approach, which in chemotherapy is translated as pharmacogenomics. This research field investigates the way genetic makeup influences a patient’s response to various drugs with the aim to minimize trial-and-error associated with drug administration. The paper introduces the role, advances and challenges of pharmacogenomics, highlighting the importance of Big Data mining to reveal the mechanisms behind drug–gene pair interaction for better patient outcomes. International consortiums have prioritized their focus on the clinical implementation of pharmacogenomics while tackling the challenges ahead: data standardization, ethical aspects and the education of physicians and patients alike to comprehend the power of pharmacogenomics to transform medical oncology.

## Plain language summary

Drug-gene interaction and implications for medical oncology

Pharmacogenomics is a research field that investigates the way the genetic makeup influences a patient’s response to various drugs with the aim to minimise trial-and-error associated with drug administration. This overview of state-of-the-art research introduces the role, advances, and challenges of pharmacogenomics, highlighting the importance of Big Data mining to reveal the mechanisms behind drug-gene pair interaction for better patient outcome.

## Introduction

**Figure 1.**
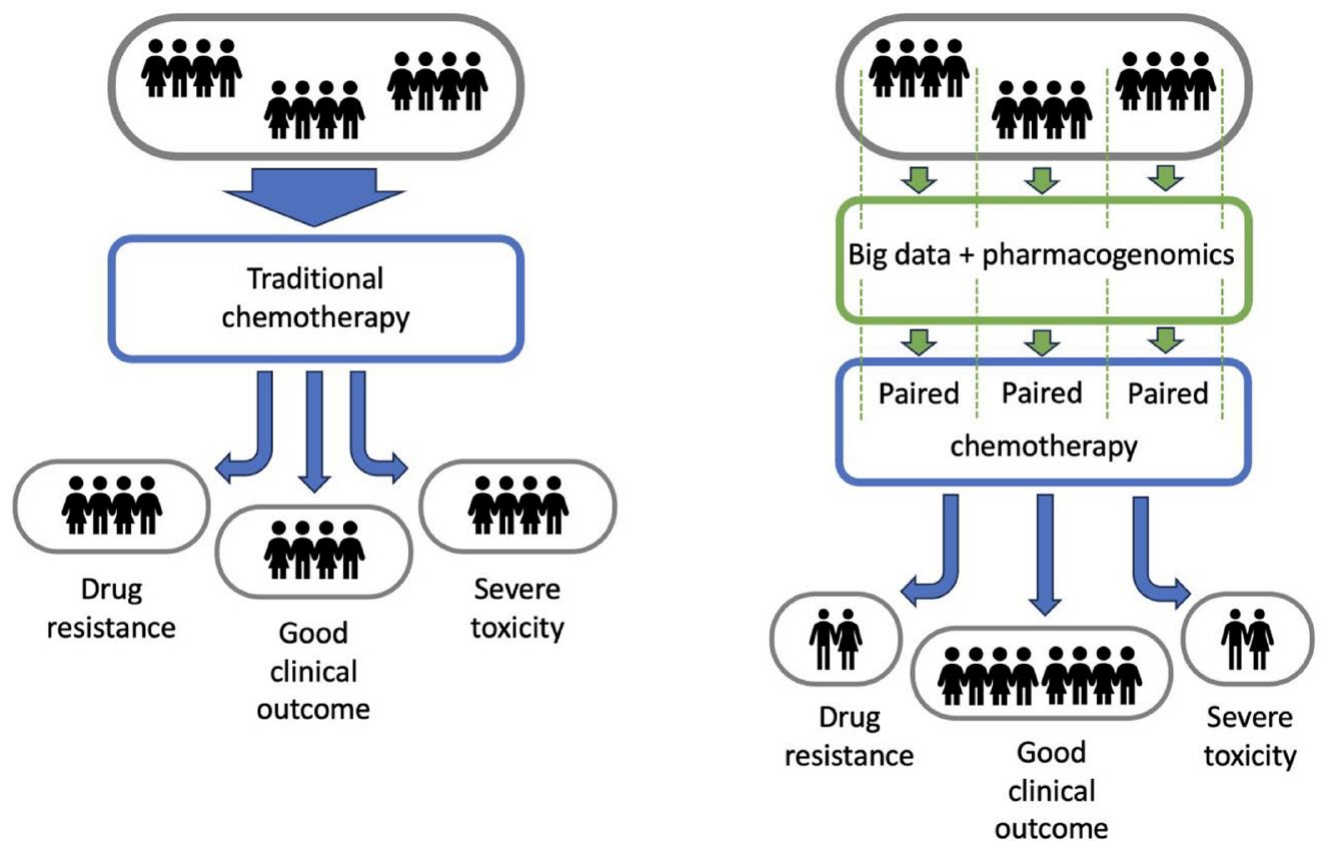
Treatment personalization using big data mining and pharmacogenomics.

Medical oncology encompasses all drug-related therapies used in cancer management. Next to surgery and radiation therapy, medical oncology is a critical component of the therapeutic process for all malignancies, particularly for systemic, disseminated disease, when surgery and/or radiotherapy are not feasible options.^1^ Conventional chemotherapy is the oldest branch of medical oncology, followed by immunotherapy and targeted therapies, the latter being more target-oriented therapies. Yet, conventional chemotherapy is still the dominant component of medical oncology, being a constituent element of most solid and haematological cancer treatments. Drug-based therapies are associated with several shortcomings that often limit their administration, thus hindering treatment success. Single drug resistance or cross resistance to drug combinations, severe acute and late adverse effects, normal tissue toxicity caused by drug–radiation interaction, are some of the major factors contributing to poor outcomes in cancer treatment. Over the years, the outcome of clinical studies revealed that there is always a subgroup of patients with severe adverse effects and/or sub-optimal treatment response, while another subgroup achieves long-term control without major toxicities. Research into these challenges showed that particular genes and gene variants are responsible for specific drug resistance and also for severe normal tissue toxicity in various cancer types. A new research field was therefore born to investigate the associations between genes and response to drug therapy to (1) develop a more fine-grained approach to treatment according to the patients’ genetic makeup and eliminate the one-size-fits-all method, (2) stratify patients based on genetic evidence in clinical trials and (3) minimize trial-and-error associated with drug administration (Figure 1). Pharmacogenomics (often interchanged with pharmacogenetics which mainly refers to genetic causes of individual rather than multiple genetic variations in drug response) is a highly promising field that can tackle the aforementioned chemotherapy-related challenges by using comprehensive drug and gene databases and applying algorithms for finding correlations between drug–gene pair interactions.

This narrative review aims to present the role and advances in pharmacogenomics, as well as the challenges regarding the implementation of the field into healthcare settings to facilitate clinical decision making towards a patient-tailored treatment. The role of Big Data mining is also discussed in the context of medical oncology. There are a number of international pharmacogenomic consortiums that currently work towards data standardization to enable pharmacogenetic data sharing across electronic healthcare record systems to expedite the implementation of pharmacogenomics into clinical practices. Education of clinicians and patients alike plays a crucial role in the successful and timely employment of pharmacogenomics in healthcare in general, and medical oncology in particular.

## The role and shortcomings of chemotherapy in cancer management

Chemotherapy entails the administration of cytostatic or cytotoxic drugs that are either cell cycle-specific or have no specificity towards a particular phase of the mitotic cycle. This characteristic is important for combined therapies when the effect of chemotherapy is expected to aid the effect of radiotherapy, most often in the form of cellular sensitization (see Table 1). Drugs differ in biochemical structure, molecular mode of action, pharmacology, clearance and side effects. All these characteristics render them adequate for a particular anatomical site or histopathology. Different drug classes exhibit different cytotoxic mechanisms and their interaction with radiation is expected to enhance the anti-tumour effect of radiotherapy.

**Table 1.** Common chemotherapeutic agents used in combination with radiotherapy for the management of solid tumours.

Drug class	Representative agents	Cell cycle effect/properties	Commonly targeted cancers	References
Alkylating agents	Carboplatin, Cisplatin Chlorambucil, Cyclophosphamide Dacarbazine, Melphalan, Oxaliplatin, Temozolomide	DNA adduct, cell arrest in G2 phase, angiogenesis inhibitor, hypoxic cell sensitizer	Lung, head and neck, breast, ovarian, sarcoma, multiple myeloma, liquid cancers	2–4
Antimetabolites	5-Fluorouracil, Cytarabine, Gemcitabine, Hydroxyurea Methotrexate, Pentostatin	Interference with DNA and RNA synthesis	Head and neck, breast, ovarian, gastrointestinal, leukaemia	5–7
Antibiotics (including anthracyclines)	Doxorubicin, Epirubicin Bleomycin, Mitomycin-C, Mitoxantrone	Interference with cell growth; Hypoxic cell cytotoxin; DNA damage	Wide-ranging cancers	8, 9
Nitrosoureas (similar to alkylating agents)	Carmustine, Lomustine Semustin, Streptozocin	Inhibit DNA repair; they can cross the blood–brain barrier thus are useful for brain tumours	Brain	10, 11
Topoisomerase inhibitors (plant alkaloids)	Irinotecan, Topotecan Etoposide, Teniposide	Interference with enzymes that assist with DNA replication; cell arrest in the radiosensitive phases of cell cycle	Lung, ovarian, colorectal, gastrointestinal, pancreatic, leukaemias	12, 13
Mitotic inhibitors/Taxanes (plant alkaloids)	Docetaxel, Paclitaxel Vinblastine, Vincristine Vinorelbine	Inhibition of microtubule function for cell replication in all phases of the cell cycle; inhibit mitotic division	Breast, lung, liquid cancers	14, 15

**Figure 2.**
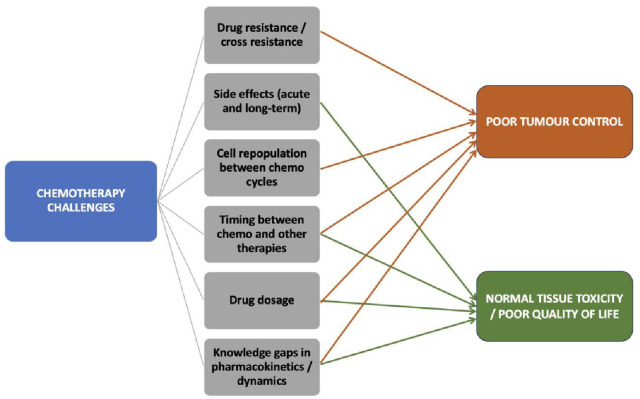
Challenges posed by chemotherapy and their effect on patients’ outcomes.

Chemotherapeutic agents have seen significant developments over the last few decades,^16^ yet there are several ongoing challenges that limit their efficacy in solid tumours: optimal dosage, drug resistance and cross resistance to multiple agents, side effects (some being irreversible), optimal timing between chemotherapy and other treatments (surgery, radiotherapy, immunotherapy, etc.), repopulation between cycles of chemotherapy and the lack of complete understanding of the pharmacokinetics and pharmacodynamics of a specific agent (Figure 2). Of the above challenges, resistance to chemotherapeutic agents and normal tissue toxicity are perhaps the most common obstacles to achieving optimal results. To prevent poor tumour response to a specific agent induced by drug resistance, chemotherapeutic agents from different drug classes (see Table 1) are often used together in so-called cocktails. A downside of multi-agent chemotherapy is the increased normal tissue toxicity, which combined with radiation-induced toxicity can often lead to severe adverse effects. Usually, different drug classes cause different side effects, while all affect rapidly proliferating cells irrespective of the cell’s status (i.e. healthy or malignant). Additionally, alkylating agents were shown to increase the risk of leukaemia (5–10 years post-therapy),^17^ anthracyclines can cause permanent cardiac lesions when administered in high doses,^18^ topoisomerase inhibitors increase the risk of second cancers,^19^ while taxanes are linked to increased risk of nerve damage.^20^

Outcome analysis of clinical trials conducted over the last decades has demonstrated improved survival with concurrent chemoradiotherapy, however, they could not show differences in results as a function of drug dosage, drug timing/sequencing or type of chemotherapy.^21,22^ While developments in radiation delivery techniques have led to increased dose conformality and better sparing of adjacent critical organs, the case is not the same with chemotherapy, where drug-related side effects still represent a dose-limiting factor. Moreover, the combination of chemotherapeutic agents with radiotherapy often causes additional adverse effects through drug–radiation interaction, an area with several lacunae that requires further research for effective solutions. The sub-optimal results obtained by trials with certain drug–radiation combinations originate from deficient/lack of preclinical evidence or inadequate translation of preclinical results into clinical settings.^23,24^ Based on the current status of systemic drug therapy, it is considered that progress in chemotherapy has reached a plateau that will not be overcome unless research directions are reoriented towards personalized and biomarker-guided therapeutic approaches.^25^

What the outcome of clinical trials has shown us is that in most instances there exists a subgroup of patients that responds optimally to therapy with minimal toxicity while another category of patients exhibits debilitating side effects and/or poor tumour control, despite the strict eligibility criteria employed by the trial. The fact that cancer patients with the same histopathological and immunohistochemical parameters have different treatment outcomes, raises an important issue regarding patient stratification and treatment personalization. To progress in the field of individualized therapy it is critical to identify all patient- and clinical-related parameters that influence response to treatment (such as chemotherapy) and create databases with the collated information to be made available to all interested researchers for further processing and interpretation.

An evolving research area that aims to personalize drug therapy based on the patients’ individual genetic characteristics is pharmacogenomics. Since the birth of the field in the 1950s, pharmacogenomics has seen substantial growth owing to the completion of the Human Genome Project. Clinical applications in this area are widespread throughout all medical specialties, with oncology representing an important candidate for future developments.

## Pharmacogenomics – the key to personalized chemotherapy

In cancer management, pharmacogenomics might play a key role in adapting chemotherapy to a patient’s genetic makeup, dealing with the multitude of acute and long-term side effects that occur due to both expected and unforeseen reactions to chemotherapeutic agents. Furthermore, owing to the cocktail-type administration of cancer drugs for better tumour response, a large range of healthy tissues is affected, making the management of side effects more difficult. As already mentioned above, the key to research advances in personalized chemotherapy is the identification of patient-specific factors that are the source of inter-patient variability in terms of treatment response. In this context, it is important to mention two large precision medicine clinical trials: NCI-MATCH (National Cancer Institute – Molecular Analysis for Therapy Choice) and NCI-ComboMATCH that both aim to match the therapeutic drug with the genetic properties of the individual. Thus, patients accrued by these trials are allocated a therapy based on the genetic changes identified in their cancer cells through genomic sequencing, with the primary goal of determining the objective response rate and progression-free survival/side effects as secondary aims.^26,27^ The two trials aim to examine targeted, rather than conventional drugs, either FDA approved or currently tested in other trials provided they have demonstrated effectiveness against cancers that present with a specific genetic alteration. Unlike the NCI-MATCH trial, which focuses on single drug testing, the NCI-ComboMATCH trial evaluates combinations of targeted drugs that are supported by preclinical in vivo evidence. A key goal of this latter trial is to overcome drug resistance to single-agent treatment, based on genomic-specific targeting with combined agents. Results of NCI-MATCH trial arms have already been published for a number of drugs/cancers, with the latest reports summarized in Table 2 showing mixed outcomes among which some warrant further investigations.

**Table 2.** Summary of drug-cancer matching studies for personalized therapy based on the NCI-MATCH trial.

Drug/reference	Cancer type/characteristics	Trial results/observations
Erdafitinib^28^	Cancers with FGFR1–4 mutations or fusions	Primary endpoint (objective response rate) was met in patients with pretreated solid tumours; erdafitinib showed effectiveness in patients with FGFR-altered cancers outside of currently approved indications.
Trastuzumab/Pertuzumab^29^	HER2-amplified cancers (non-breast/gastroesophageal)	The combined drug was active without meeting the set efficacy goal. Further research into resistance pathways and HER2 targeting strategies is justified.
Osimertinib^30^	Cancers with EGFR mutations (T790M or rare activating)	Primary endpoint was not met for efficacy; responses were observed in neuroendocrine and epithelial carcinoma with rare EGFR mutations.
Palbociclib^31^	Cancers with CCND1, 2 or 3 amplification and expression of the retinoblastoma protein	Palbociclib was not effective at treating non-breast solid cancers with a CCND1, 2 or 3 amplification; further research is not warranted with palbociclib as single agent.
Trametinib^32^	Cancers with NF1 or GNA11/Q-mutations	Primary endpoint was not met for efficacy; yet, significant responses and prolonged stable disease in some disease subtypes warrant further investigation.

CCND, cyclin D; FGFR, fibroblast growth factor receptor; EGFR, epidermal growth factor receptor; GNA11, guanosine nucleotide-binding protein alpha-11 gene; NF1, neurofibromatosis 1.

It is expected that advances in DNA technology such as next-generation sequencing (or massively parallel sequencing – a high-throughput DNA sequencing that enables the evaluation of the entire genomic sequence of a patient through parallel processing), will revolutionize cancer genomics and facilitate an improved understanding of carcinogenesis through the identification of new driver genes and mutagenetic patterns.^33,34^

Genetic research is undergoing in the field of conventional chemotherapy as well, focusing on two major aspects of chemotherapy effects: normal tissue toxicity and drug resistance. There is an arsenal of cancer-specific genetic alterations and pathways that have been correlated with resistance to various drugs, while other genetic mechanisms still need elucidation for their role in drug resistance.^35^ Similarly, for several chemotherapeutic agents, the culprit genes for severe toxicity have been identified and measures are underway to update treatment protocols with genotype testing requirements for treatment guidance and adaptation. For instance, in case of 5-fluorouracil administration (see Table 1 for common cancers treated with this agent) research showed that toxicity induced by this drug is largely linked to deficiency in a key metabolic enzyme – dihydropyrimidine dehydrogenase (DPYD). A meta-analysis of individual data originating from 7365 cancer patients has confirmed the correlation between variants of DPYD and severe toxicity in patients treated with fluoropyrimidines either as sole agents or combined with other drugs and/or radiotherapy.^36^ To illustrate the role of pharmacogenomics in personalized chemotherapy, a prospective, multi-centre, drug safety analysis was conducted on 1103 cancer patients planned to start on fluoropyrimidine-based chemotherapy regimen.^37^ Outcome analysis showed higher rates of severe toxicity in DPYD variant carriers than in wild-type patients (*p* = 0.0013), suggesting a 50% dose reduction to limit severe adverse effects.

In a number of cancers, the accumulation of several polymorphic gene variants was connected to high-grade systemic toxicities and often to elevated risk of disease progression.^38,39^ Innovative in-depth studies into drug resistance and toxicity rely on novel high-throughput technology, such as next-generation sequencing, to identify functional defects associated with poor outcomes and explore in parallel multiple mechanisms that are responsible for high-grade toxicities in some patients and those that control drug resistance in others.

Genetic testing should, therefore, become a prerequisite for personalized treatment. Clinical implementation initiatives of pharmacogenomics include two types of testing approaches: reactive and pre-emptive. Within reactive testing, the patient undergoes testing for a particular gene or groups of genes after making the medical decision regarding the drug to be administered. Thus, the implementation of reactive models is conducted on a gene-by-gene basis, with tests being completed when the need arises for high-risk drug prescriptions.^40^

Pre-emptive (or proactive) testing models allow genotyping results to be available before any prescribing decision, thus enabling genomic variants to be regarded as inherent patient characteristics during treatment planning.^41^ Proactive testing is therefore gradually replacing the reactive testing model to make pharmacogenomic testing results as well as dosing recommendations available pre-emptively in electronic health records (EHRs).

An important aspect concerning the outcome of personalized drug therapy is the patient’s genetic background with respect to drug metabolism. Inherited capabilities of drug metabolism and transport are encoded in the cytochrome P450 (CYP450) enzymes which were shown to predict chemotherapy outcome.^42^ CYP450 enzymes play a key role in the oxidative metabolism of drugs, influencing drug activation and clearance, and are largely expressed in the liver. Latest research has identified the genetic polymorphism in CYP450 genes to play an important role in a number of cancers regarding drug pharmacokinetics, suggesting the clinical relevance of CYP450 genotyping for personalized therapy.^42^ In view of the above, research interest is already shown in gene therapy involving genetically engineered CYP enzymes to be incorporated into cancer cells in order to enhance the conversion of prodrug into its active metabolite.^43^ This approach is believed to reduce drug-related side effects through dose optimization and improvement of toxic by-product breakdown.

## Big Data in medical oncology

Big Data is a term that has been in use since the late 1990s to indicate a collection of data that is ‘too large for traditional tools and approaches’. The above definition is intentionally vague as the continuous development of technology constantly moves the line where something would be considered ‘too large’. In its broader meaning, Big Data encompasses not only the data itself but also the methods used to analyse and extract information from the data. It is easy to think about Big Data just as an overgrown regular database, when in fact Big Data sets are a collection of structured, semi-structured and unstructured data. In medical oncology research, the portion of data that is unstructured is quite significant, as much of the useful information comes in the form of EHRs. Herein lies the challenge of interpreting this information to extract parameters that can be used in advanced statistical methods.

The main characteristic of a Big Data set is its volume of information. Interesting to note that, historically speaking, information has always existed, but data has not, as the information was not being recorded. The digital age has brought with it the capturing and storage of many new aspects of healthcare, from personal monitoring to fitness devices. The volume of recorded data is increasing at a rapid pace not only because of the addition of these new sources but also due to the ever more detailed capturing of information from traditional sources, creating a pool of data that is larger and also more fine-grained in its representation of the information. Another aspect of a Big Data set is its variety in type and nature. Structured data like lab results are part of a dataset just like semi-structured data (e.g. medical images stored in a structured database but representing an unstructured source of information). A valuable and rich source of information is the EHRs storing text-based information inputted by many healthcare professionals over a long period of time for each patient, thus representing a completely unstructured source of data. Also to be considered is the velocity of incoming data into a Big Data set. Studies carried out on datasets are usually using a snapshot of the data so they treat it as a static assembly of information, but in reality, the influx of new data never stops, meaning that the storage infrastructure has to be able to continuously accept new data.

**Figure 3.**
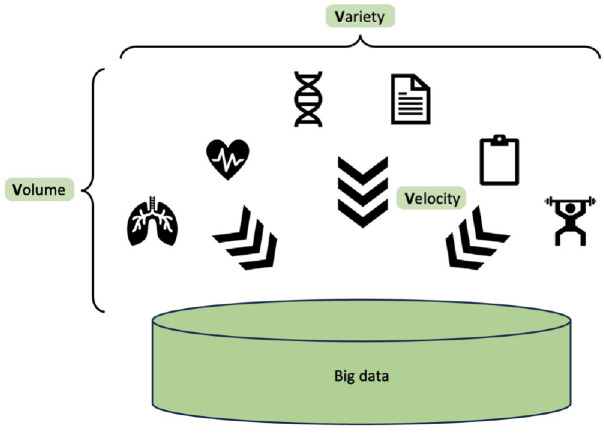
The original three V-s of big data: volume, variety and velocity.

The above-described volume, variety and velocity are the original three V-s of big data introduced by Doug Laney^44^ in a Meta Group research publication in 2001 (Figure 3). Since then, other characteristics have been added to more comprehensively describe a Big Data set, like value (the significance of the stored information in relation to the dataset goals), veracity (the accuracy and reliability of information) and variability (the irregularity of the information structure due to the wide range of sources).

Considering the presented characteristics of a Big Data set, it becomes obvious that one of the main attributes of a Big Data project is the storage infrastructure. Traditionally, the relational model of a database has been in use for information storage, presenting a relatively easy-to-understand concept and a well-defined way of interaction via the Structured Query Language (SQL) language. The advent of Big Data sets has exposed the limitations of relational databases that adhere strictly to the ACID transactions principle.^45^ This schema-oriented model is expecting information in a structured manner according to the defined schema, which is not always the case in a Big Data set featuring variable sources of information. The volume of data is also creating issues for relational databases which are able to scale only vertically by adding more computing power to the fixed infrastructure housing the database. The velocity of the incoming data for a Big Data set completes the three V-s that make it clear that a parallel infrastructure able to scale horizontally on a multitude of devices or the cloud is more suited for the job.

A new concept of database called NoSQL is able to overcome these limitations. This model does not have a set schema and is able to scale over large computing infrastructures. For example, the key-value store concept present in NoSQL is akin to a dictionary where a unique key is associated with a value. This model is lending itself to parallel operations distributed over large infrastructures. The name NoSQL initially stood for ‘non-SQL’ to describe the fact that these databases did not expose an SQL interface, but recent developments have warranted a change to ‘not only SQL’ to show that they might support SQL-like interfaces. The scalability over relatively cheap infrastructure is a great feature, but some applications have been missing the transactional guarantees of the ACID principles absent in the NoSQL model. A novel type of database called NewSQL has been developed to incorporate the scalability requirements with the transactional necessities of modern applications.

Storage is only one aspect of a Big Data project. The data needs to be processed in an efficient way and given its volume, variety and velocity, it requires a highly parallelized system to achieve this. A very popular solution is the use of the open-source Hadoop framework with its distributed file system running on commodity hardware.^46^ This system organizes the infrastructure into nodes that can serve as either storage or computing units or both. The processing of data follows the MapReduce programming model in which at the mapping step the computing tasks are distributed to the nodes in the system and the reducing step is represented by the aggregation of results from each node.

New drug discovery has traditionally been done via in vivo animal studies which is an expensive and complex procedure. The process is a hit-or-miss affair with some studies showing that 9 out of 10 drug candidates fail between phase I trials and regulatory approval.^47^ The potential of computational modelling using Big Data repositories has been recognized by researchers as a way to simplify and speed up new drug discovery.^48^ Using artificial intelligence methods such as machine learning and deep learning, complex correlations can be discovered hidden deep within Big Data sets. Outcome predictions can suggest the drug candidates most likely to achieve their goals, allowing researchers to channel their resources only towards new drugs with a high chance of success. In vivo studies are still needed to demonstrate efficacy as the high complexity of living organisms is almost impossible to emulate in a computational environment, meaning that in silico success is not a guarantee of in vivo effectiveness.

To enable computational modelling research, several initiatives have created Big Data sets that are publicly available to both add and download data. In the United States, the PubChem database is a vast collection of over 118 million chemical compounds, 317 million substances and over 1.6 million bioassays providing an excellent source of target response information.^49^ In the United Kingdom, ChEMBL^50^ is a manually curated database of bioactive molecules with drug-like properties, holding over 2.4 million compounds tested against 15,000 targets. The Canadian DrugBank^51,52^ initiative is an online database containing information on drugs and drug targets. DrugMatrix^53^ is one of the world’s largest toxicogenomic sources of information, allowing researchers to efficiently create a toxicological assessment and thus shorten the time needed to formulate a xenobiotic’s potential for toxicity. Other notable drug databases are the Pharmacogenomics Knowledge Base (PharmGKB) with its affiliated Clinical Pharmacogenetics Implementation Consortium (CPIC)^54,55^ and the Therapeutic Target Database,^56^ just to list the largest ones.

Genetic databases for cancer research encompass data in the form of genes, variants, phenotypes or other gene products that are specific for diverse malignancies. Gene databases allow operations such as gene data mining, disease genome sequence analysis and knowledge-driven variant interpretation.^57,58^ A comprehensive human gene database GeneCards enables navigation through the pool of genes, proteins, diseases and biological pathways, inferring direct and indirect scored associations between thousands of variant genes and disease phenotypes.^57^ Many of such genes and variants that play key roles in regulating signalling pathways or maintaining malignant phenotypes are already identified as potential therapeutic targets in oncology.

Given the available clinical databases, a natural step in overcoming long-term or permanent normal tissue toxicity caused by chemotherapy is the analysis of Big Data to find correlations between the administered treatment and patient response. A database that is an ideal source for data mining for personalized drug therapy and a valuable resource for therapeutic biomarker discovery in cancer cells is the Genomics of Drug Sensitivity in Cancer (GDSC).^59^ GDSC is the largest public database encompassing information on drug sensitivity of cancer cells and molecular markers of drug response.^60^ To date, the database contains information on 1000 human cancer cell lines that were screened with 621 compounds targeting 24 pathways. Furthermore, the database includes over 576,000 dose–response curves and 722,000 genomic associations that underwent testing, providing a platform for new discoveries of therapeutic biomarkers.

An example of a tool that enables the identification of new therapeutic targets from differentially expressed genes as well as drug candidates is TARGETgene, a software that integrates resources from a number of public databases and allows the exploration of target genes and validation of predictions via user-defined benchmark genes and curated cancer-specific genes.^61^ This tool enables the construction of a whole genome network, integrating heterogeneous data at the genomic and proteomic level, based on which the software identifies potential therapeutic targets using network metrics. The platform also provides a list of existing drugs and agents that might have an effect on the identified targets, including the option of drug repurposing if containing gene-matching compounds.

To assist in uncovering druggable genes and particularly gene fusions (resulting from chromosome rearrangements) that have been identified across numerous cancer types, a computational framework was developed to facilitate the elaboration of therapeutic strategies for patients exhibiting the fusion.^62^ The computational approach, FusionPathway, aims to elucidate pathway-dependent resistance mechanisms to fusion-targeting therapies and to find solutions to overcome treatment resistance, through a domain-based network approach to infer the molecular interactions and pathways correlated with a specific gene fusion.^62^

Over the last couple of decades, machine learning tools have been widely employed in genetic research to shed more light on the impact of genetic mutations on response to drug therapy. The evidence-based pairing of drug–gene aimed to minimize normal tissue toxicity, to increase cytotoxic efficiency in tumours and to overcome drug resistance, has become an essential component of personalized chemotherapy through developments in pharmacogenomics research. Computational methods developed to predict drug response in preclinical models mainly focus on selection of molecular properties that affect drug response and quantification of response to drug therapy, using machine learning techniques.^63^ Artificial neural networks for drug response prediction have been used since the 1990s, based on the assumption that metabolic characteristics of tumour cells reflect the intrinsic cellular response to chemotherapeutic agents (whether sensitivity or resistance).^64^ Through a neural network trained as a non-linear regression tool to predict a continuous function of chemosensitivity, a pre-treatment distinction was made between drug-resistant and drug-sensitive glioma cell lines using the metabolic profiles of the cells as indicated by proton nuclear magnetic resonance spectra.

Deep neural networks have currently been developed to predict drug response from genomic data. The Cancer Drug Response profile scan (CDRscan) developed by Chang et al.^65^ is a deep learning model that uses large-scale drug screening assays containing genomic profiles originating from 787 human cancer cell lines and 244 structural drug profiles to predict response to chemotherapeutic drugs. The model uses a two-step convolution architecture, with the genomic signature of cell lines and the molecular drug characteristics being individually processed and then merged by virtual docking. CDRscan was applied to 1487 approved drugs and 37 new drugs (both oncologic and non-oncologic) with potential in cancer treatment. Goodness of fit analysis between observed and predicted drug response displayed high prediction accuracy (*R*^2^ > 0.84, Area Under Curve > 0.98), the model showing potential not only for drug selection based on patients’ genomic profile but also for predicting the feasibility of drug repurposing.

A convolutional neural network model of drug responsiveness (DeepIC50) developed to address high dimensional features (27,756) integrates both genomic profiles and molecular descriptors of three drug classes from large datasets (GDSC) with the aim to predict IC50 (the half maximal inhibitory concentration of a drug) in gastric cancer patients.^66^ Prediction of drug responsiveness as indicated by the model was comparable to responsiveness observed in human gastric cell lines, indicating the applicability of the model for novel drug predictions, as well as for other cancers.

Based on the assumption that similar chemicals exhibit similar effects, Choi et al.^67^ have developed the Reference Drug-based Neural Network built on multiple ElasticNet regressors to exploit a set of drugs with known characteristics (reference drugs) and using a benchmark for classifying and evaluating new (untrained) drugs. Using the similarities between the reference and untrained drugs, the model predicts drug sensitivity for specific drug-cell line pairs with high accuracy, while also offering indications on drug repurposing.

The future of big data analysis in pharmacogenomics probably relies on further developments in deep learning methods, given their advantage over conventional machine learning algorithms of feature learning from raw and noisy data. The recent success of deep learning models in drug response prediction and drug repurposing is due to the growth of raw data in drug and genomic research as well as the advances in computer science that enabled handling large amounts of data via powerful graphical processing units.^68^ While several limitations are yet to be overcome, deep learning techniques will see great developments in the near future through optimization of the methods involved, to increase their predictive power.

## Implementation of pharmacogenomics into clinical settings

### Current status

The aim of modern drug research is the implementation of pharmacogenomics into clinical settings. Initiative in this direction have been launched by several institutions throughout the United States, Europe and Asia.^41,55,69,70^ One of the latest, single-institution reports on the clinical implementation of pharmacogenomics is a study from the University of Colorado, where research was conducted to enable a pre-emptive return of clinical pharmacogenomic results using a research biobank. Their goals were to facilitate the employment of pharmacogenomic results originating from biobanks in patient care, to evaluate how the results of pre-emptive genotyping can be returned to the EHR for future clinical decision making, and also finding ways to educate clinicians about the implementation of pharmacogenomics into clinics.^71^ Some other single institution initiatives towards the implementation of pharmacogenetics/pharmacogenomics include: the customized pharmacogenetics genotyping array for personalized medicine programmes implemented by the University of Florida/Stanford University,^72^ the CLIPMERGE PGx programme of Mount Sinai Medical Center which is a clinical initiation of personalized medicine through EHRs and genomics/pharmacogenomics,^73^ the 1200 Patient Project initiated by the University of Chicago that offers pre-emptive pharmacogenomic testing during outpatient care,^74^ the University of Maryland personalized anti-platelet pharmacogenetics programme,^75^ the PG4KDS protocol from St Jude Children’s Research Hospital^76^ and one of the most recent European projects, PriME-PGx from La Princesa University Hospital, Madrid, Spain.^77^

There are also large-scale initiatives to encourage implementation of pharmacogenomics research results in clinical practice. The CPIC in the United States^78,79^ and the Ubiquitous Pharmacogenomics programme in Europe^69,80,81^ are two extensive projects that aim to establish a healthcare system that will allow effective treatment optimization based on pharmacogenomics results.

The CPIC was established in 2009 as a shared project between PharmGKB and the National Institute of Health (NIH) to overcome one of the greatest obstacles in clinical pharmacogenomics: the translation of genetic testing results into actionable prescribing decisions for a specific drug. Their key aim was to create, curate and distribute freely available, evidence-based and peer-reviewed clinical practice guidelines regarding drug–gene interaction to facilitate bench-to-bedside translation of knowledge.^78^ Until 2020, CPIC has published 23 guidelines encompassing data on 19 genes and 46 drugs, covering various therapeutic areas.^79^ The visibility and recognition of the Consortium’s research progress over the past decade has led to a widespread use of their resources, and international application of their guidelines, being recognized as the gold standard source for clinical implementation of pharmacogenetics. An important aspect considered by the Consortium is the development of prioritization guidelines, for establishing which pharmacogenetic tests and drugs should undergo clinical implementation based on levels of actionability assigned to drug–gene pairs. Furthermore, through regular updates, CPIC provides the latest information on evidence concerning drug–gene interaction (whether actionable or requiring testing) as well as guidelines for clinical practice regarding specific drug–gene pairs. Standardization, another key aspect within pharmacogenomics, is addressed by the collaborative approach between CPIC and experts in the field from various organizations to develop consensus terms for pharmacogenetic phenotypes and allele function.^82^ The Term Standardization Project led to the successful adoption by the scientific community of the established terms, and they are incorporated into the CPIC guidelines. Additionally, the standard terms will enable pharmacogenetic data sharing across electronic healthcare record systems to facilitate clinical decision making and expedite the implementation of pharmacogenomics into clinical practices.^83^

The Ubiquitous Pharmacogenomics Consortium has been funded by the European Commission through the Horizon-2020 programme with its major goal to determine the collective clinical value of implementing a panel of pharmacogenomic markers into routine clinical care, given that previous research focus was mainly on specific drug–gene pairs.^69^ According to van der Wouden et al. ‘pharmacogenetic panel-based testing represents a new model for precision medicine’. Motivated by the need of assessing the cost-effectiveness of a pharmacogenomic approach based on the panel of available markers that have the ability to guide drug therapy, the Ubiquitous Pharmacogenomics Consortium initiated the PREemptive testing for Prevention of Adverse drug REactions (PREPARE) study.^84^ To meet the major goal of the study, that is, enabling the quantification of the collective clinical utility/value of pharmacogenomics markers within one trial for pharmacogenomics-guided drug therapy (via dose and drug selection) across multiple actionable drug–gene interactions, the project aims: (1) to define a composite primary endpoint facilitating quantification of the anticipated effect (this is represented by relevant adverse reactions caused by the index drug associated with a drug–genotype pair); (2) to avoid overrepresentation of frequently prescribed drugs within the patient sample; (3) to design the strategy to be applicable across various healthcare settings and ethnicities and (4) to design statistical analysis to avoid dilution of effect by initially excluding patients without gene–drug interaction from the analysis.^84^ PREPARE is a multi-centre, randomized study conducted in seven European countries, with participant countries being randomized to start with either pharmacogenetics-guided prescribing or standard of care (control arm) for 18 months, after which a new set of patients was accrued, and the opposite strategy implemented for another 18 months (6944 eligible patients). The PREPARE trial is the first prospective clinical study that investigated the effect of pharmacogenomics guidance using a pre-emptive panel of 12 genes, generating evidence for personalized therapy, while also providing a framework for future studies.^85^

An observational study designed and initiated by the Mayo Clinic aimed to recruit a large patient cohort (over 11,000) for pre-emptive pharmacogenomic testing through the Right Drug, Right Dose, Right Time: Using Genomic Data to Individualize Treatment Protocol (RIGHT protocol).^86^ The source cohort for the RIGHT protocol study was the Mayo Clinic Biobank which encompasses biological specimens from 57,000 subjects. The RIGHT study aims to develop the electronic healthcare record infrastructure to provide point-of-care clinical decision support and to evaluate the effect of clinically integrated proactive testing on treatment outcomes, while assessing the following scientific aspects: (1) the association between genetic variants and clinical outcome in patients undergoing drug therapy with a known interaction with these genes; (2) the impact of pre-emptive testing on patient outcome and healthcare costs; (3) the clinical outcomes associated with pharmacogenomic variants of unidentified clinical significance and (4) the impact of pharmacogenomic testing on practitioners and patients’ perspectives alike.^86^

To facilitate multidisciplinary research towards personalized therapy based on genomic information, the NIH has organized and funded a US consortium encompassing medical research institutions to connect electronic medical records with genetic data. The Electronic Medical Records and Genomics (eMERGE) network brings together a multidisciplinary team of experts from genomics to clinical medicine and from informatics to statistics to cover all aspects involved in both fundamental research and clinical implementation.^87^ The key aim of the consortium is to combine biorepositories with electronic medical record systems for the employment of genomic medicine in clinical settings, thus to create a data-driven method of healthcare delivery. Their research underwent several phases of development, currently the focus being on the assessment of health impact, cost-effectiveness as well as ethical and social implications.

### The challenges ahead

For a more efficient use of the Big Data available in biobanks and for their timely translation into clinical settings an aspect that requires attention is the education of healthcare providers. Several reports have demonstrated that there is still a large percentage of healthcare professionals that are only vaguely- or not familiar with pharmacogenomic testing and only a small number of providers embraced genomic testing and use it as part of their clinical routine. The results of a survey conducted among US healthcare providers in paediatric fields revealed that over 71% of responders were unfamiliar with pharmacogenomics despite the fact that half of them reported prior education and training in the field.^88^ Only 26.4% of healthcare providers reported recent genetic testing requests for their paediatric patients. Very similar results were reported by other groups interested in the manner pharmacogenetic testing in the United States is perceived by paediatric providers, indicating that only 24% of survey responders have ordered such tests and that 90% of healthcare providers are not comfortable ordering and interpreting results without the assistance of a geneticist or pharmacogenomic expert.^89^

A European survey of healthcare professionals has found that while 84% of responders consider pharmacogenomics as being relevant to their practice, one-third only ordered a pharmacogenomic test in the year prior to the survey.^80^

A systematic review of the literature completed to assess the provider experience with clinical pharmacogenomic information has found 33 such studies conducted throughout the world involving physicians and pharmacists alike.^90^ Obstacles in the expanded use of pharmacogenomics in clinics were identified in the form of (1) underdeveloped clinical decision support infrastructure, (2) lack of third-party payer coverage policies and reimbursement and (3) lack of/limited clinician and patient understanding of the role played by genomic testing in personalized treatment.^90^

These survey results clearly demonstrate the existence of a barrier regarding the widespread clinical implementation of pharmacogenomic testing, suggesting the need for continuous education and training of professionals to recognize the value of such techniques in clinical decision making, in recommendations based on the information obtained and to acknowledge their critical role in improving patients’ life.

To illustrate the power of pharmacogenomics, Shah et al. conducted a study on 34,424 patients who received drugs for various conditions, against data available in the Partners Healthcare Biobank (‘Partners Biobank’) assessing adverse drug events. Results showed that patients with actionable pharmacogenomic results from the biobank and who received relevant medication exhibited predictable side effects.^91^ It was suggested that these adverse effects could have been avoided if the respective patients’ pharmacogenomic results were available in the EHRs.

While cost evaluations exist for a number of gene–drug pairs.^92,93^ evaluations of cost-effectiveness of complex pre-emptive models are scarce and need to be addressed within a short time to provide information to policymakers that in turn can decide on embracing and reimbursing pharmacogenetic testing and encourage further research.^94^ A comprehensive literature analysis was undertaken in the field of medical oncology to appraise the economic value of pharmacogenetic testing for cancer drugs with clinically relevant drug–gene associations, using drug classifications according to PharmGKB^®^ database (Stanford University) and the Medline/Embase databases for the literature reporting on cost-effectiveness or cost-minimizations studies.^93^ Within the 35 studies meeting the above criteria, some of the most common chemotherapeutic agents: fluoropyrimidine, irinotecan, carboplatin, cisplatin, 6-mercaptopurine, erlotinib, gefitinib, cetuximab, panitumumab and trastuzumab were assessed for their pharmaco-economic value using drug–gene relationships (and their association with dosage, adverse effects, efficiency and pharmacokinetics) based on level of evidence (high, moderate). Results lacked conclusiveness due to the mixed evidence concerning the value of genetic testing for cancer treatment guidance and owing to the fact that pharmacogenomics testing is a dominant strategy in only a limited number of studies. The authors recommend greater adherence to best practice guidelines when conducting cost-effectiveness studies.

With respect to the use of artificial intelligence in processing the available Big Data, there are opportunities for further developments and improvement of existing algorithms. While machine learning and deep learning techniques are highly suitable for identifying statistical associations in large datasets, deeming them an ideal option for the association of drug–gene pairs for various cancer types, research in this field is still scarce.

## Final thoughts

Trends in cancer treatment are heading towards precision medicine for subgroups of patients that share similar tumour features and a more personalized therapy for the individual patient based on genetic particularities. While radiomics shows potential in treatment response monitoring and outcome prediction, pharmacogenomics shows prospects in the management of chemotherapy-related side effects through better correlation between the patients’ genome and optimal choice of drug type and dosage. The availability of Big Data through clinical cancer registries, genetic/nucleotide sequence databases and the use of machine learning tools to process and analyse a large amount of data will ultimately enable the integration of pharmacogenomics into routine clinical care. Until then, a number of challenges must be overcome in the field of medical oncology that includes critical aspects for treatment success (Table 3).

**Table 3.** Challenges to overcome in medical oncology via pharmacogenomics.

Challenges	Role of pharmacogenetics in solving the challenge	Ongoing research/research aims
Drug resistance	To identify genes and variants that are responsible for specific drug resistance to single- and multi-agent therapy.	To further develop gene databases for data mining and processing of information regarding drug–gene variant compatibility. Continue accrual in the NCI-ComboMATCH precision medicine clinical trial.
Drug-induced normal tissue toxicity	To identify genes/groups of genes and variants that are responsible for severe chemo-toxicity in specific patient subgroups.	Continue accrual in the Precision medicine clinical trial NCI-ComboMATCH aiming to match therapeutic drugs with the genetic properties of the individual.
Drug–radiation interaction	To analyse drug–radiation interaction mechanisms for the avoidance of cross-toxicity and sub-optimal tumour response.	To obtain reliable preclinical evidence based on in silico, in vitro and in vivo evaluation of drug–radiation interaction, followed by well-designed clinical trials.

NCI, National Cancer Institute.

Upcoming precision medicine clinical trials must focus on conventional drugs and novel targeted therapies alike, both as sole agents and in combination with other treatment approaches. While targeted agents are becoming more common in medical oncology, the arsenal of conventional chemotherapeutic agents is prevalent enough to warrant continued thorough research to increase their safety and treatment efficacy and to overcome the plateau that medical oncology is facing vis-à-vis advances in chemotherapy.

International consortiums have prioritized their focus on the clinical implementation of pharmacogenomics. Challenges ahead include data standardization, the ethical aspect of the use of gene databases and the education of both physicians and patients to understand the impact of pharmacogenomics on personalized therapy in medical oncology (Table 4).

**Table 4.** Implementation of pre-emptive pharmacogenomic testing in clinical settings: advantages and limitations.^95–97^

Advantages	Limitations
• Better compliance, thus higher efficacy • Improved safety, thus diminished adverse events • Possible dose escalation without additional side effects • Enhanced quality of life • Reduction in number of hospitalization and/or emergency department visits • Cost-effective healthcare.	• The pool of drugs with actionable pharmacogenomic biomarkers is limited • Varied impact as a function of the variant allele frequencies in candidate genes among populations from various geographical regions • More difficult assessment in case of cocktail administration (as it is the case in most chemotherapy patients) • Challenges in decision making when alternative drug therapies are lacking/limited • Lack of worldwide availability of testing • Lack of uniform worldwide regulations/legislation on genetic testing • Lack of patient education (understanding of pharmacogenetic testing) • Patient privacy concerns • Concerns regarding potential misuse of genetic data.

Currently ‘personalized treatment’ refers to an increasingly fine-grained patient stratification to eliminate unfavourable gene–drug pairs and increase the chance of successful outcomes with statistically favourable treatment options. However, the number of deterministic factors in personalized therapy for cancer is more complex and extends beyond cellular factors, requiring a system-based approach instead of single cell/pathway-based.^98^ Environmental/epigenetic as well as physiological changes over time confer a dynamic gene expression pattern which ideally should be monitored in cancer sufferers to adjust or better tailor the required therapy. For instance, liquid biopsies are becoming valuable tools in identifying a broad range of biomarkers that offer not only diagnostic information but allow for continuous tracking of the dynamic changes occurring in tumours as well as treatment response monitoring.^99^

The utopian aim of personalized treatment is the ability to create an adjusted treatment plan for every single individual based on a multitude of individual markers/features/properties that are either already available in the patient’s medical records or obtained at the time of treatment planning. Ultimately, we aim to narrow the gauge of stratification until we can achieve a category of one, meaning that the conclusions reached are valid and particular for the single patient in question, thus achieving true personalized treatment.

## Declarations

**Ethics approval and consent to participate:** Not applicable.

**Consent for publication:** Not applicable.

**Availability of data and materials:** Data sharing not applicable – no new data generated.
